# Supplementary material for: The interdependent network of gene regulation and metabolism is robust where it needs to be
Source: Nat Commun. 2017 Sep 14;8:534. doi: 10.1038/s41467-017-00587-4 (PMC5599549; doi:10.1038/s41467-017-00587-4)
Supplement: Supplementary file 1 — Supplementary Information [file 41467_2017_587_MOESM1_ESM.pdf]

Description of Supplementary Files

File Name: Supplementary Information
Description: Supplementary Figures, Supplementary Tables, Supplementary Notes and Supplementary Reference

File Name: Peer Review File
Description:

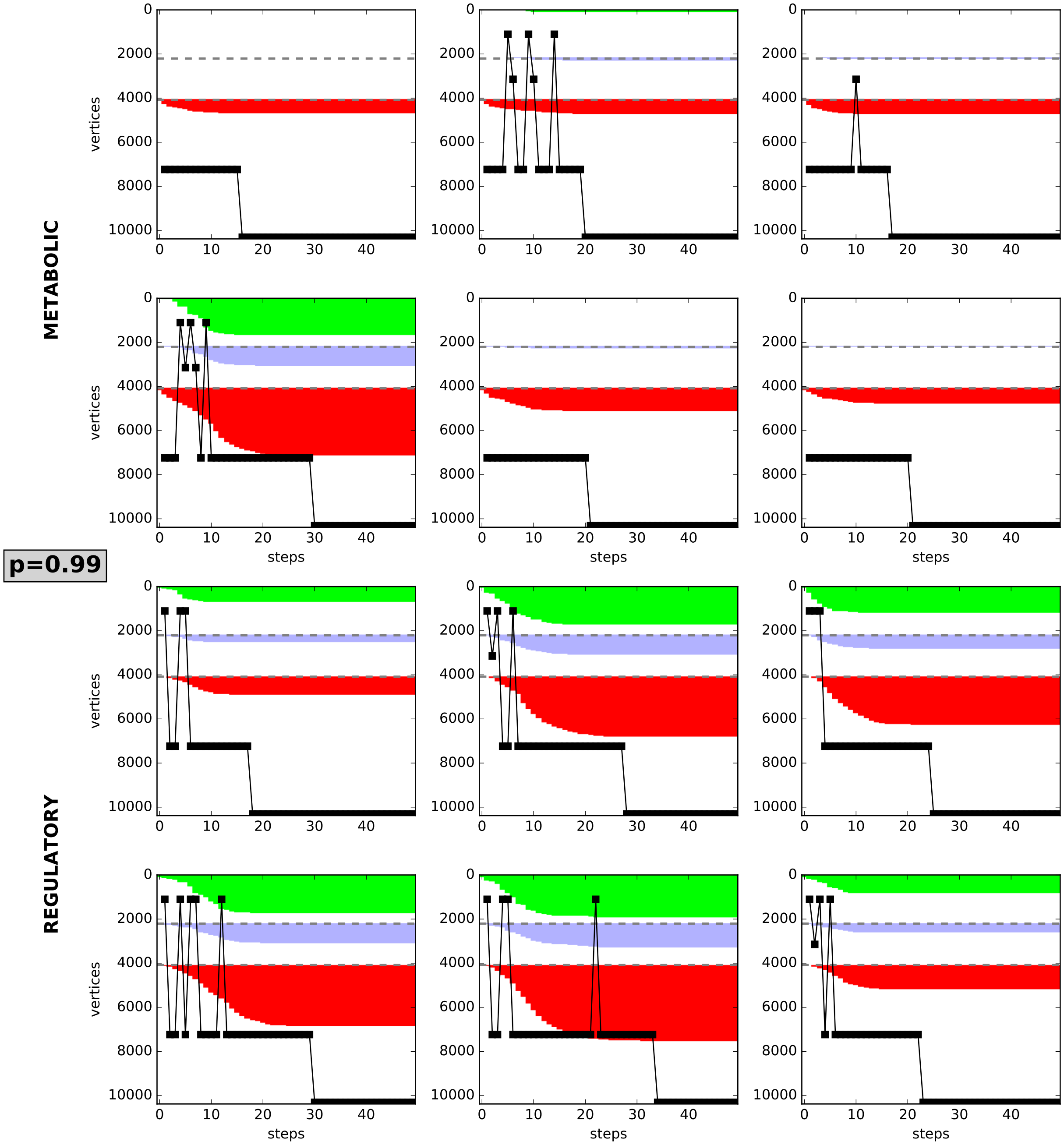

Supplementary Figure 1. **Sample trajectories of the integrative *E. coli* network.** Six sample trajectories for perturbations of size $q = 0.01$ in the metabolic (top two rows) and regulatory domain (two bottom rows), respectively. Compare to Fig. 3 in the main text.

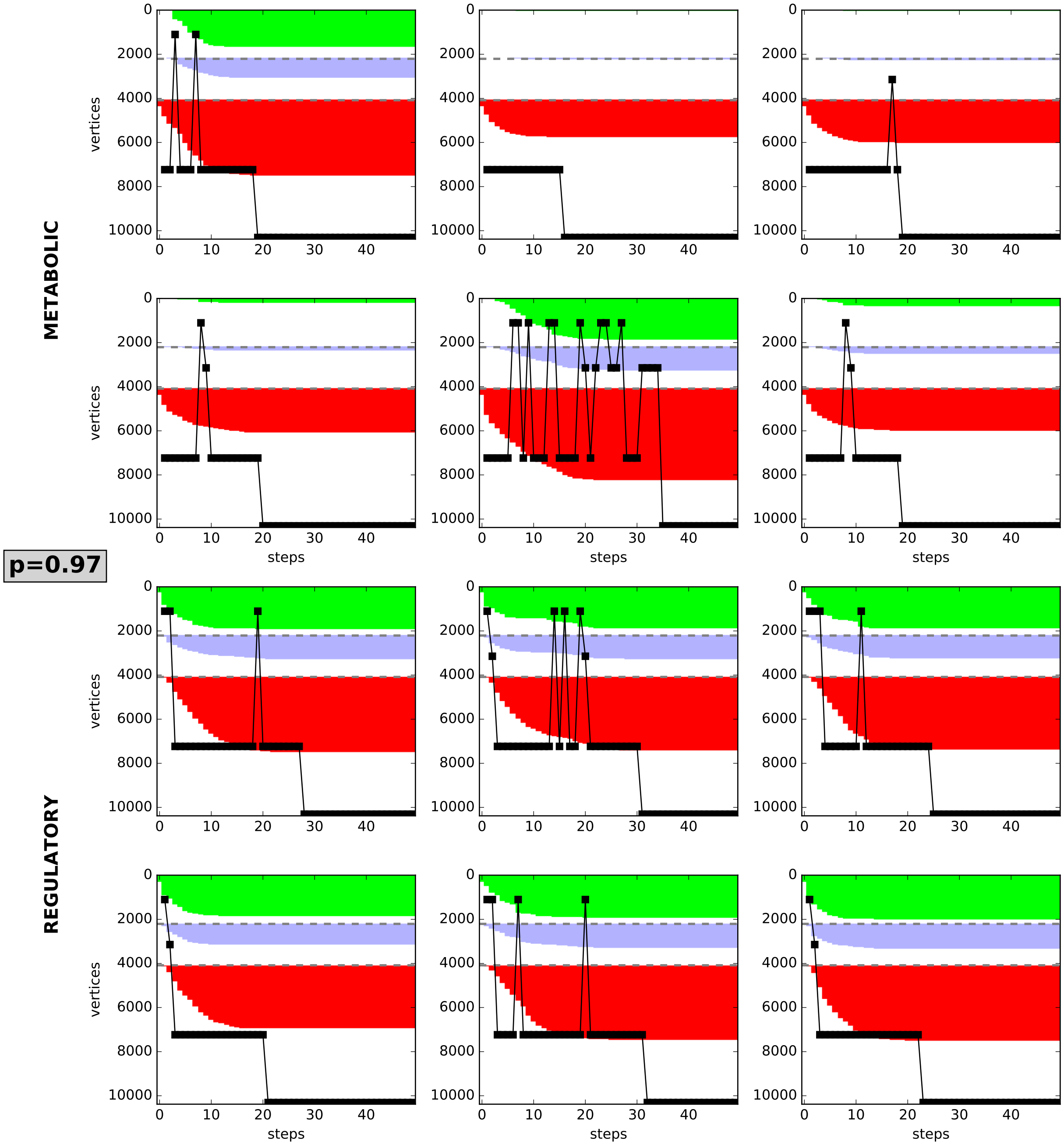

Supplementary Figure 2. **Sample trajectories of the integrative *E. coli* network.** Six sample trajectories for perturbations of size $q = 0.03$ in the metabolic (top two rows) and regulatory domain (two bottom rows), respectively. Compare to Fig. 3 in the main text.

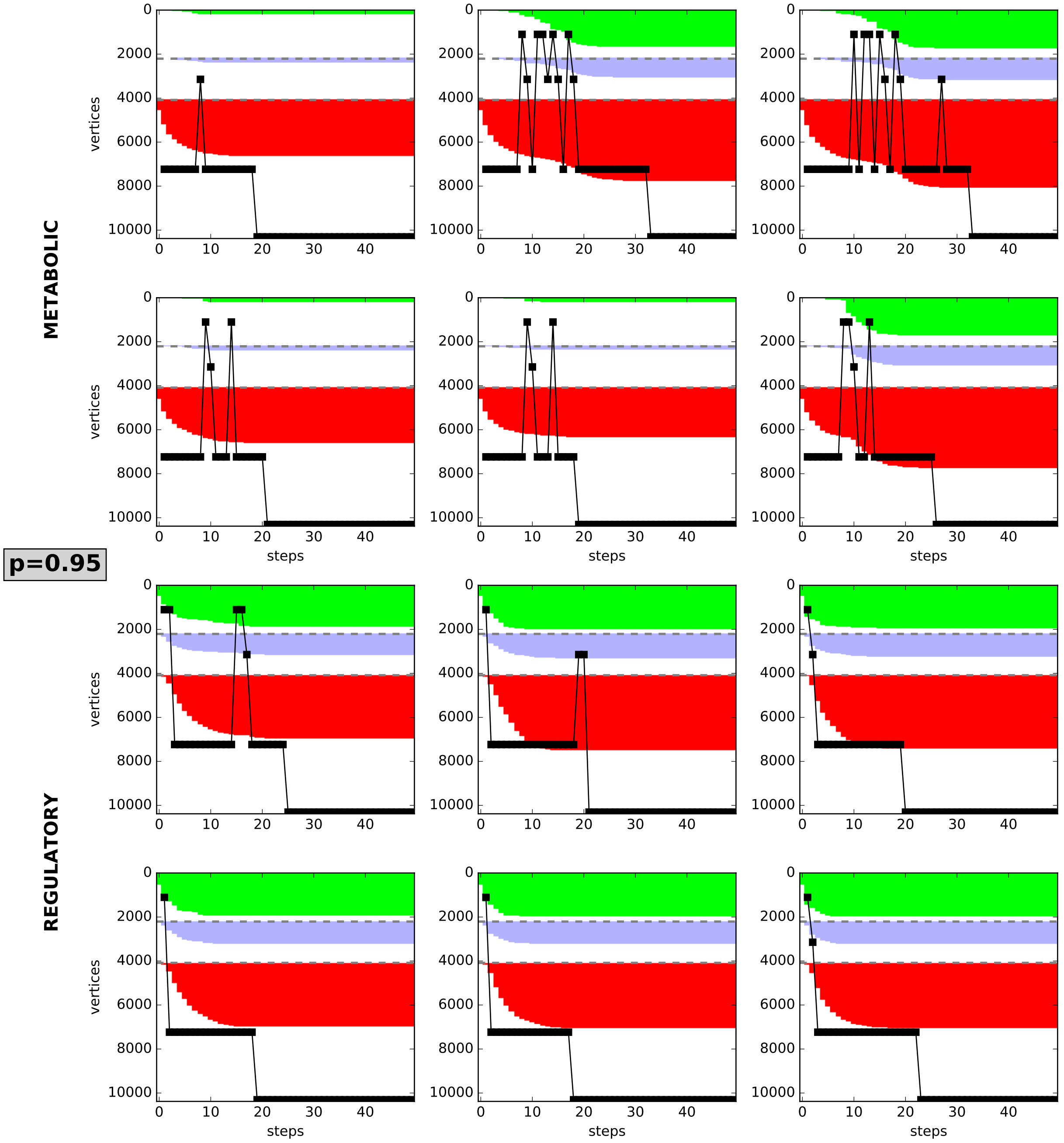

Supplementary Figure 3. **Sample trajectories of the integrative *E. coli* network.** Six sample trajectories for perturbations of size $q = 0.05$ in the metabolic (top two rows) and regulatory domain (two bottom rows), respectively. Compare to Fig. 3 in the main text.

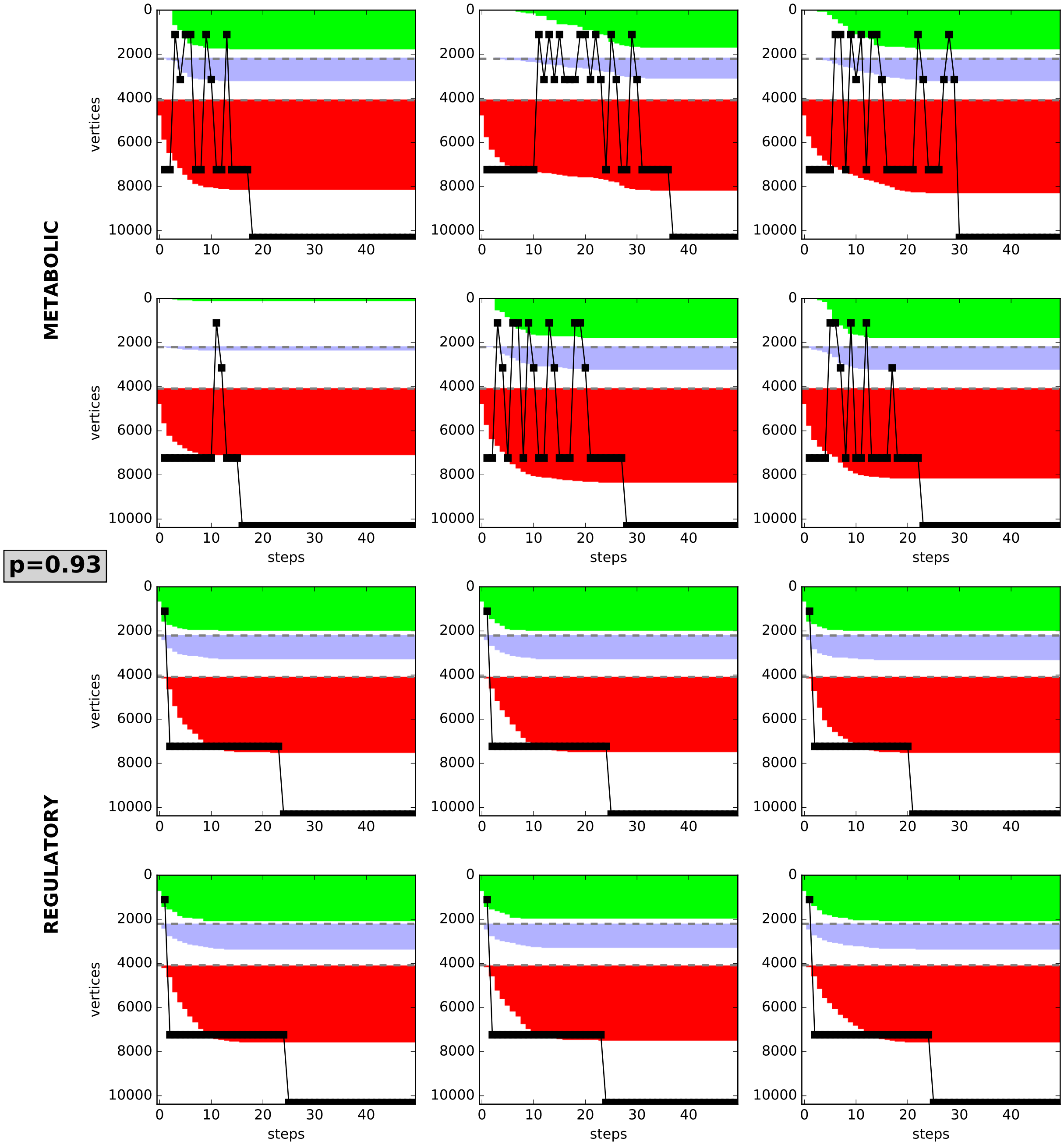

Supplementary Figure 4. **Sample trajectories of the integrative *E. coli* network.** Six sample trajectories for perturbations of size $q = 0.07$ in the metabolic (top two rows) and regulatory domain (two bottom rows), respectively. Compare to Fig. 3 in the main text.

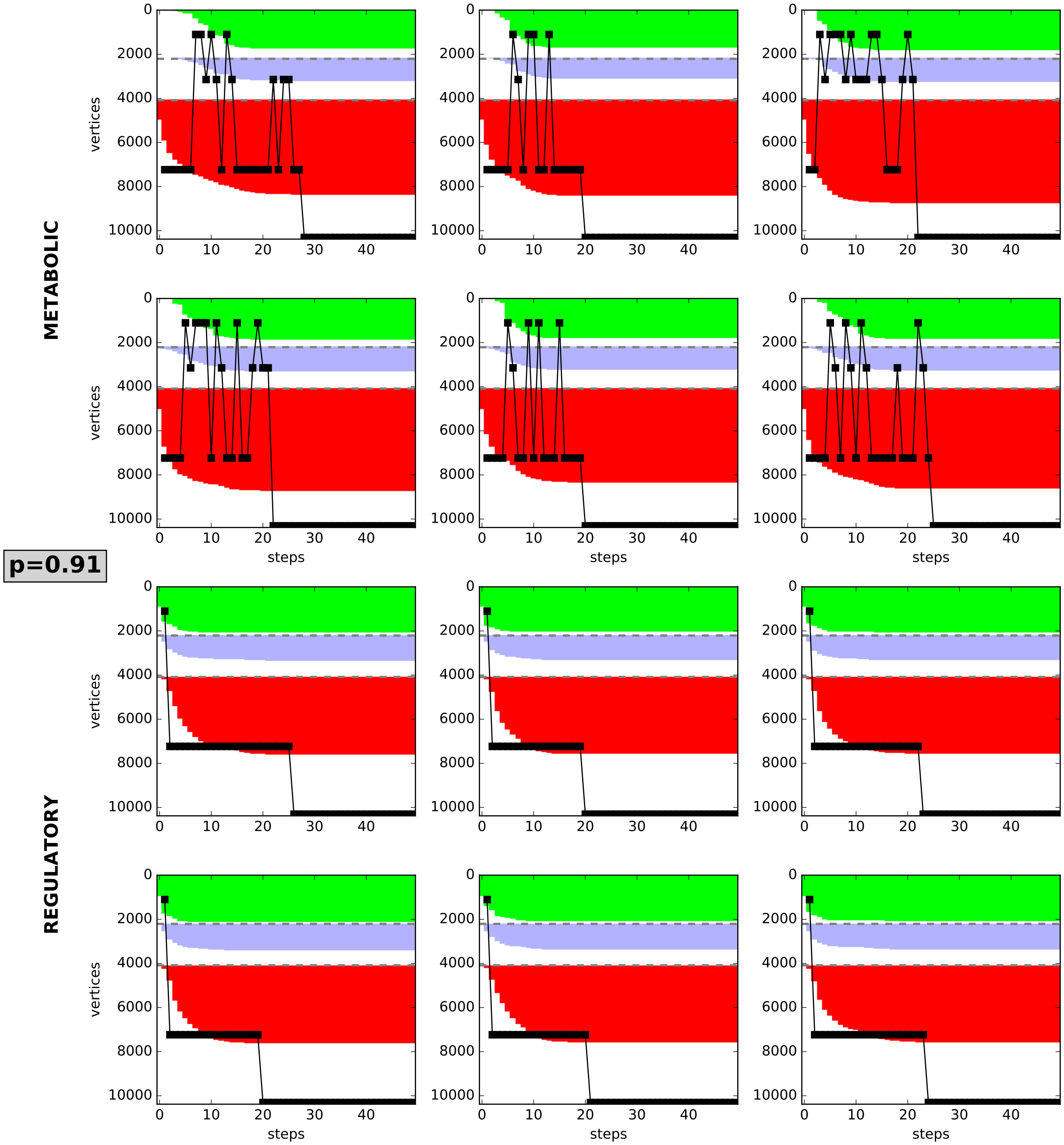

Supplementary Figure 5. **Sample trajectories of the integrative *E. coli* network.** Six sample trajectories for perturbations of size $q = 0.09$ in the metabolic (top two rows) and regulatory domain (two bottom rows), respectively. Compare to Fig. 3 in the main text.

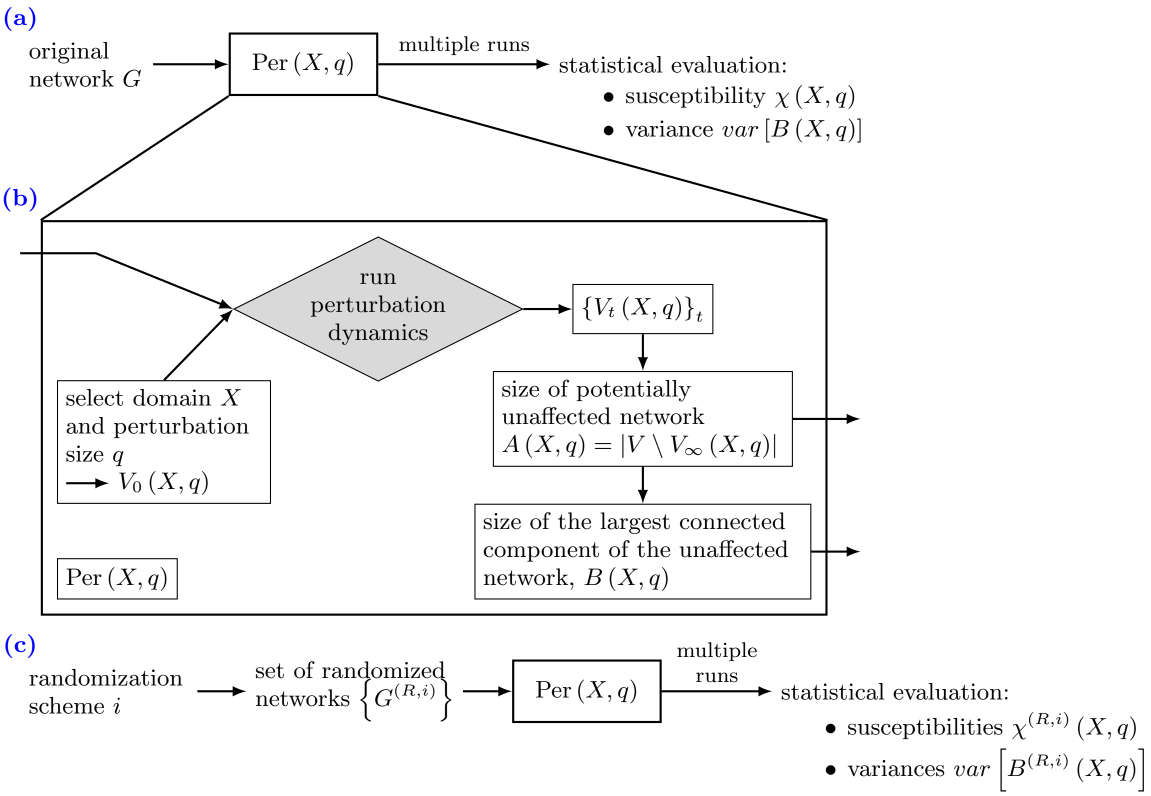

Supplementary Figure 6. **Schematic representation of the network response to localized perturbations analysis.** (a) The graph G enters the perturbation algorithm Per(X,q) characterized by the domain X and the size of the perturbation q; statistics over multiple runs are then evaluated in terms of susceptibilities and variances; (b) details of the perturbation algorithm; based on X and q the set $V_0(X,q)$ of initially perturbed vertices is randomly selected; running the perturbation dynamics leads to a time series of affected vertices, $\{V_t(X,q)\}_t$, which is subsequently evaluated yielding the size of the unaffected network (which can be derived from the number of vertices in the set $V_t(X,q)$ in the asymptotic regime) and the relative size of the largest (weakly) connected component of the unaffected network, $B(X,q)$; (c) same as (a), but for randomized networks.

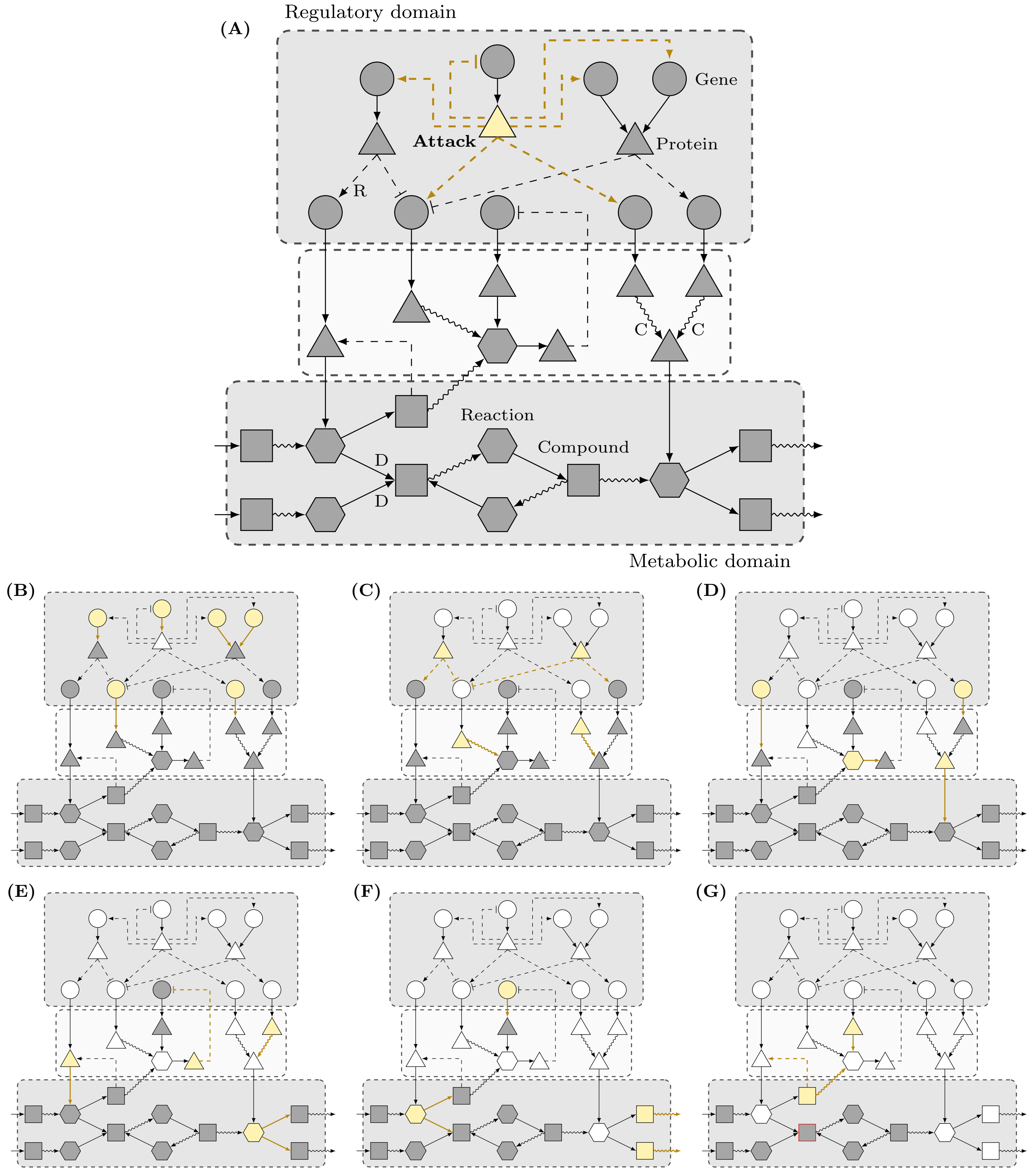

Supplementary Figure 7. **Sketch of the propagation dynamics of a single-vertex perturbation.** Sketch of a single-vertex perturbation (Attack) and its propagation according to the percolation model (equations (1) and (2), main text) in a sample three-domain organized network, with regulatory domain (top), (protein-) interface (middle) and metabolic domain (bottom). The process shown in frames (A-G) is identical to the one shown in compressed form in Fig. 2 in the main text but presents more detail concerning the biological characteristics of the system. Here the vertices are distinguished according to the BCVs, namely, gene (○), protein (△), compound (□) and reaction (⬡) while the arrow line styles denote in the same way the three logical categories of edges: C – curly, D – solid, and R – dashed. The newly affected vertices in each step are highlighted in yellow whereas the ones previously switched off are white. The orange colored edges denote the spread of the next time step. A vertex that has been reached but remains unaffected is framed red.

Supplementary Table 1. **Overview of the four custom-built randomization schemes in order of their strictness.** In particular, the degree of freedeom is denoted by the possible pairs of edges available for the randomization and by the conserved quantities of the graph: source domain (SD), target domain (TD), logical category of an edge (LCE), biological category of a vertex (BCV), and biological category of an edge (BCE).

Scheme	Possible pairs	Conserved quantities
DOMAIN	$134,942,137$	(SD, TD)
DOMAIN_LCE	$59,075,210$	(SD, TD), LCE
DOMAIN_BCV	$54,592,007$	(SD, TD), BCV
DOMAIN_BCE	$42,774,454$	(SD, TD), BCE

SUPPLEMENTARY NOTE 1

Description and Schematic Overview of the analysis

The network response to localized perturbations analysis presented here (see Supplementary Fig. 6), is a multi-step method entailing several runs of the perturbation algorithm, $\mathrm{Per}(X,q)$, and the statistical evaluation of these runs. More precisely, for each analysis 500 runs of the perturbation algorithm have been performed and the set of the remaining vertices have been evalueted, e.g., in terms of the susceptibility, $\chi\left(X,q\right)$ (part (a) in Supplementary Fig. 6).
For a single run of the perturbation algorithm, $\mathrm{Per}(X,q)$, a domain X has to be chosen where a perturbation of size $q = 1 - p$ (measured as a fraction of vertices) will be applied,

$$X \in \{R, I, M, T\}$$

with R – regulatory domain, I – protein interface, M – metabolic domain, T – total network.

Based on X and q the set of initially perturbed vertices, $V_0\left(X,q\right)$, is randomly selected. The state of the system can also be described as a vector of Boolean state variables, $\boldsymbol{\sigma}$,

$$\sigma_i \in \{0,1\}\,,\; i = \{1,\ldots,|G|\}$$

where 0 denotes a perturbed vertex and 1 an unaffected one.
Running the perturbation dynamics described in the main text and also illustrated in Supplementary Fig. 7 will (probably) cause the failure of further vertices resulting in a time series of affected vertices, $\{V_t(X,q)\}_t$ (or, equivalently, to the trajectory $\boldsymbol{\sigma}(t,\,X,q)$). The size of the affected network after t propagation steps can be described as

$$|V_t\left(X,q\right)| = |G| - \sum_{i=1}^{|G|} \sigma_i\left(t,\,X,q\right)$$

From the set of affected vertices in the in the asymptotic regime, $V_\infty\left(X,q\right)$, the size of the unaffected network, $A\left(X,q\right)$, and the size of the largest (weakly) connected component (LCC) of the unaffected network, $B\left(X,q\right)$ are computed (Supplementary Fig. 6, part (b)),

$$\begin{aligned} A\left(X,q\right) &= \left|V \setminus V_\infty\left(X,q\right)\right|, \\ B\left(X,q\right) &= \left|\mathbf{LCC}\left[V \setminus V_\infty\left(X,q\right)\right]\right|. \end{aligned}$$

Randomized networks (we used sets of 500 instances for each of the randomization schemes) can be passed to the algorithm instead (Supplementary Fig. 6, part (c)).

Supplementary Table 2. **Overview of plotted quantities and parameter values.** Summary of the plotted quantities and parameter choices in the Figs. 3–5 in the main text as well as in the Supplementary Figs. 1–5.

Figure	Quantity plotted	Parameter values
	**Main Text Figures**	
3	$V_t(X,q)$ (or $\boldsymbol{\sigma}(t,\, X, q)$) as a function of time t	a,c: $X = R$, b,d: $X = M$<br>a,b: $q = 0.01$, c,d: $q = 0.03$
4	$\chi^{(R,i)}(X,q)$ as a function of $p = 1 - q$	i in ['unshuffled', $1, 2, 3, 4$] (top to bottom),<br>X in $[T, R, M]$ (for each frame)
5	$A(X,q)/N$ as function of $p = 1 - q$	$X \in \{M, R\}$, $i \in \{$'unshuffled', $1, 2, 3, 4\}$
	**Supplementary Figures**	
1	$V_t(X,q)$ (or $\boldsymbol{\sigma}(t,\, X, q)$) as a function of time t	$X = M, R$; $q = 0.01$
2		$X = M, R$; $q = 0.03$
3		$X = M, R$; $q = 0.05$
4		$X = M, R$; $q = 0.07$
5		$X = M, R$; $q = 0.09$

SUPPLEMENTARY NOTE 2

Notation of vertex and edge categories

The following notation concerning vertices $v_i \in V$ and edges $e_k \in E$ and their properties have been used. In our integrated network model a vertex is characterized by its biological function yielding seven unique biological categories of a vertex (BCVs), 'reaction' (rxn), 'compound' (cmp), 'gene' (gn), 'protein monomer' (pm), 'protein-protein complex' (ppc), 'protein-compound complex' (pcc), and 'protein-rna complex' (prc; see Supplementary Table 3),

$$v_i^{\mathrm{BCV}} \in \{rxn, cmp, gn, pm, ppc, pcc, prc\}.$$

Introducing an additional vertex classification facilitates the assignment to one of the three functional domains as well as the edge characterization. The domain-related categories of a vertex (DCVs) are eight-fold: 'gene' (g), 'protein' (p), 'complex' (x), 'enzyme' (z), 'reaction' (r), 'compound' (c), 'educt' (e), 'product' (d),

$$v_i^{\mathrm{DCV}} \in \{g, p, x, z, r, c, e, d\}.$$

While the categories g and r are a one-to-one translations of the corresponding BCVs, i.e., gn and rxn, the domain-related category c only comprises vertices of BCV cmp but the inverse does not hold. The remaining five categories are ambiguous assignments for both BCVs to DCVs and vice versa. The complete mapping of BCVs onto DCVs is given in Supplementary Table 4.

An edge is characterized by its source and target vertices, $v_s^{(k)}$ and $v_t^{(k)}$, and their corresponding domains ('source domain', SD and 'target domain', TD), as well as the edge's logical category and its biological category; an edge is given by

$$e_k = (v_s^{(k)}, v_t^{(k)})\,,\; v_j^{(k)} \in \{G_R, G_I, G_M\},$$

thus determining SD and TD. The logical category of an edge (LCE) determines, qualitatively speaking, whether a perturbation will propagate along this edge via a logical `AND` or a logical `OR`; the three categories are 'conjunct' (C), 'disjunct' (D) and 'regulation' (R),

$$e_k^{\mathrm{LCE}} \in \{C, D, R\}.$$

As an illustration of the potential linkages, two case examples are presented in Supplementary Fig. 8.

The biological categories of an edge (BCEs) are derived from combinations of the domain-related categories of a vertex (DCVs), plus 'transport' (t) and regulation (r^*),

$$e_k^{\mathrm{BCE}} \in \{g \to p, p \to x, c \to x, z \to r, e \to r, r \to d, t, r^*\}.$$

The mapping of biological categories of edges onto logical categories of edges is given in Supplementary Table 5.

Supplementary Table 3. **Biological categories of a vertex (BCVs) of the integrative *E. coli* network and their potential incoming logical categories of an edge (LCEs).** The target vertex category may constrain the incoming edges to be: 'conjunct' (C), 'disjunct' (D) or 'regulation' (R). The detailed vertex composition will be given in Grimbs *et al.* [1].

Vertex	BCV	LCEs
	gene (gn)	R
	protein monomer (pm)	D, R
	protein-protein-complex (ppc)	C, D, R
	protein-compound-complex (pcc)	C, D
	protein-rna-complex (prc)	–
	reaction (rxn)	C, D, R
	compound (cmp)	C, D

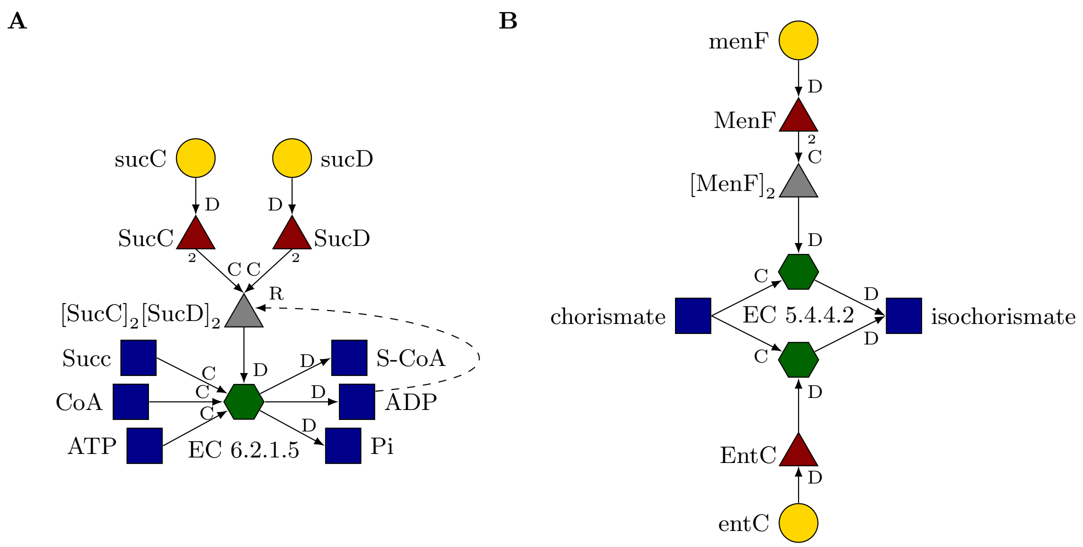

Supplementary Figure 8. **Two biological case examples of potential linkages. A** succinyl-CoA synthetase (EC 6.2.1.5) and **B** isochorismate synthase (EC 5.4.4.2). The vertices are denoted by the common biological abbreviation (see EcoCyc webpage) and the respective biological category of a vertex (BCV): ● gene, ▲ protein monomer, ▲ protein-protein-complex, ⬢ reaction, ■ compound. Moreover, the involved edges are labeled with the corresponding logical category of an edge (LCE): 'conjunct' (C), 'disjunct' (D) and 'regulation' (R). The additional numbers indicate stoichiometric coefficients for the complex formation.

Supplementary Table 4. **Domain-related categories of a vertex (DCVs) of the integrative *E. coli* network and the mapping to biological categories of a vertex (BCVs) and involved biological categories of an edge (BCEs).** The item shapes represent the BCVs: ● gene, ▲ protein monomer, ▲ protein-protein-complex, ▲ protein-compound-complex, ▲ protein-rna-complex, ⬢ reaction, ■ compound.

DCV	BCVs	BCEs
gene (g)	●	$g \rightarrow p$
protein (p)	▲, ▲, ▲	$g \rightarrow p$, $p \rightarrow x$
complex (x)	▲, ▲	$p \rightarrow x$, $c \rightarrow x$
enzyme (z)	▲, ▲	$z \rightarrow r$
reaction (r)	⬢	$z \rightarrow r$, $e \rightarrow r$, $r \rightarrow d$
compound (c)	■	$c \rightarrow x$
educt (e)	▲, ▲, ▲, ■	$e \rightarrow r$
product (d)	▲, ▲, ▲, ■	$r \rightarrow d$

Supplementary Table 5. Biological and corresponding logical categories of an edge. Biological categories of an edge (BCEs) and the corresponding logical categories of an edge (LCEs): 'conjunct' (C), 'disjunct' (D) and 'regulation' (R), for each vertex linkage of the integrative *E. coli* model. The different linkages are denoted by the combinations of BCVs: gene, protein monomer, protein-protein-complex, protein-compound-complex, protein-rna-complex, reaction, compound. The detailed edge composition will be given in Grimbs *et al.* [1].

BCEs	LCEs	Vertex linkages
gene $\rightarrow$ protein ($g \rightarrow p$)	D	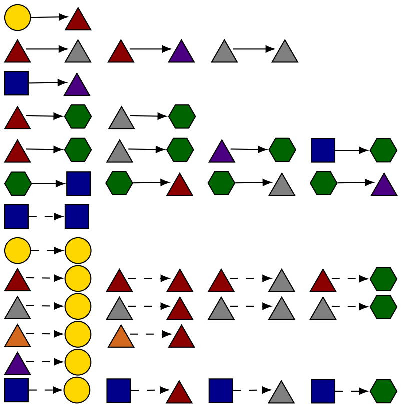
protein $\rightarrow$ complex ($p \rightarrow x$)	C	
compound $\rightarrow$ complex ($c \rightarrow x$)	C	
enzyme $\rightarrow$ reaction ($z \rightarrow r$)	D	
educt $\rightarrow$ reaction ($e \rightarrow r$)	C	
reaction $\rightarrow$ product ($r \rightarrow d$)	D	
transport (t)	C	
regulation (r^*)	R	

SUPPLEMENTARY REFERENCES

[1] Grimbs, A., Klosik, D. F., Bornholdt, S. & Hütt, M.-T. Integrative system-wide modeling of metabolic and regulatory processes in *Escherichia coli* (2017). *(In preparation).*
